# Wounds of the Intestines

**Published:** 1884-07

**Authors:** S. D. Gross

**Affiliations:** Emeritus Professor of Surgery in the Jefferson Medical College of Philadelphia


### Selections.

### WOUNDS OF THE INTESTINES.*

BY S. D. GROSS, M. D., LL. D., D. C. L. OXON., LL. D., CANTAB.,

*Emeritus Professor of Surgery in the Jefferson Medical College of Philadelphia.*

[*Medical News.*]

There are few topics in surgery that have received less attention than the nature and treatment of wounds of the intestines. Even our text-books say very little upon the subject, and that little is seldom founded upon original observation. When we consider the frequency of these lesions, their great gravity—if not almost uniform fatility, and the doubts and misgivings with which they are approached by most practitioners, it will not require any apology on my part for bringing the matter formally before this meeting.

While every other branch of surgery has been enriched by papers and monographs almost in countless numbers, the literature of wounds of the intestines is exceedingly scanty, and, in many respects, unsatisfactory. The older writers have transmitted to us nothing of value, and even as late as the close of the last and the beginning of the present century not one of our systematic writers had any fixed or settled notions upon the subject. The author who came nearest to this was Benjamin Bell, of Edinburgh, whose *System of Surgery*, in six octavo volumes, published nearly one hundred years ago, did much to diffuse a taste for surgery, not only in Scotland and in England, but on the Continent of Europe and in the United States. It is an interesting fact that the first foundation of a rational treatment of lesions of this kind was laid in this country in 1805, by Dr. Thomas Smith, of St. Croix, in his *Inaugural Dissertation*, presented to the Faculty and Trustees of the University of Pennsylvania. His experiments, twelve in number, were performed upon dogs, with a view, mainly, it would seem, of ascertaining the best mode of sewing up wounds of this description, without

* Read before the American Surgical Association, April 30, 1884.

going at all into the question of their mode of repair. Seven years after this, namely, in 1812, appeared the admirable treatise of Benjamin Travers, of London, entitled, *An Inquiry into the Process of Nature in Repairing Injuries of the Intestines.* His researches were more especially directed to the elucidation of penetrating wounds and to the proper management of the bowel in strangulated hernia —topics discussed in a very able and scientific manner. In conducting these researches, the English surgeon availed himself of experiments upon dogs, and of the ample clinical opportunities afforded him at Guy's and St. Thomas's Hospitals. I need hardly add that Mr. Travers's essay was one of the most original and important contributions made to surgical science in the early part of the century. The work was not republished in this country, and did not, I think, excite much attention even on the other side of the Atlantic. For a knowlede of its valuable contents the surgeons of Europe and America were mainly indebted to that storehouse of surgical facts, Prof. Samuel Cooper's *Surgical Dictionary*, a work of world-wide celebrity.

Among the French surgeons who in the first third of the present century busied themselves in enlarging our knowledge of the treatment of wounds of the intestines by an appeal to experiments upon the inferior animals may be mentioned, with special commendation, the name of Jobert, Lembert, Gély, Amussat and Choisy, on account of the peculiar forms of sutures respectively recommended by them.

Convinced that this department of surgery was susceptible of still further investigation, I commenced a series of experiments in 1841, which was continued, with occasional intermissions, until the end of 1843, when an account of them was published in the *Western Journal of Medicine and Surgery*, issued at Louisville, under the editorship of Drake, Yandell and Colescott. A small edition was also issued in separate form, but as the printing office in which it had been stored away was soon after consumed by fire, few copies were saved. Hence the reason why such little notice has been taken of my labors. Indeed, the only account I have ever seen of them is comprised in the *Surgical History of the War*.

The object which I had in view in undertaking these researches was, first, to ascertain the process employed by nature in repairing wounds of the intestinal tube; and, secondly—and more particularly—to determine, if possible, the best methods of treatment. The experiments, upwards of seventy in number, were performed exclusively upon dogs, as the most eligible animals that could be selected for the purpose. The wound was generally made in the small bowel, not only because it is more readily accessible than the large, but because, when injured, it is more liable to give rise to extravasation of fecal matter, and also, possibly, to high inflammation.

Gunshot injuries of the bowels, so common during the war of the rebellion, are more ably and fully discussed in the *Surgical History of the War* than in all other works put together, and what is true of wounds caused by firearms is equally true of other injuries of the intestinal canal. Dr. Otis spared no pains to embody in his chapter on this subject the latest information to be found in the magnificent medical library accessible to the Surgeon-General of the Army and his intelligent and laborious assistants, to whom the profession is so much indebted for our Army Medical Reports.

Wounds of the intestines may, like similar lesions in other structures, be incised, contused, lacerated, or punctured, the latter including those made by gunshot. Incised wounds are occasionally inflicted accidentally upon the bowel in hysterectomy and ovariotomy, and in operations performed for the relief of strangulated hernia. All injuries of this kind of the intestinal tube, whether single or multiple, simple or complicated, derive their chief importance from two sources—escape of fecal matter and peritoneal inflammation. The accompanying hemorrhage is generally a subordinate occurrence.

The manner in which wounds of the intestinal tube are repaired depends very much upon their character. Simple incised wounds, if properly treated, heal by union by the first intention, as similar wounds involving the skin, muscles and other structures; that is, the effused plasma soon becomes organized and is transformed into cicatricial tissue. When the suture is carried through the mucous membrane the healing process is more tardy, and, if some of the stitches should give way prematurely, so as to allow the edges of the wound to gap, the union will be effected mainly through the agency of the serous coat. Granulations always form with difficulty, and rarely afford much aid in filling up the breach. In nearly all of my experiments upon dogs, I noticed that the wounded bowel at the seat of the injury was speedily glued either to the omentum or to some adjacent coil of intestine, thereby forming, in most cases, an effectual barrier to fecal effusion. Conjecturally, we may assume that a similar occurrence obtains in a human subject. The ligatures, when the ends are cut close, are always discharged into the interior of the tube, the period at which this takes place varying with many circumstances from ten to fourteen days, as the ordinary average, to three or four weeks, as the extreme. Lacerated, ragged, contused, punctured and gunshot wounds heal in the same manner as incised, but the process is more tardy and more liable to fail. When wounds, of whatever kind, are left to themselves, or to the unaided efforts of nature, the subjects of them either perish from their effects, as fecal effusion, peritonitis, hemorrhage, or septicæmia, or, if they recover, their safety is due to the adhesions which the injured portion of the tube contracts with the surrounding parts.

The diagnosis of wounds of the bowel is a matter of primary consideration, as upon its prompt determination the success of our treatment must mainly hinge. The possibility of this will, of course, mainly depend upon the situation in which the bowel is found at the time of the accident. If it has escaped through the wall of the abdomen, it will generally be easy to find the injured part by the egress of some of its contents, as feces, mucus, or bile, or all these together; and so also when there is a discharge of some, or all, of these substances through the outer wound, although there be no protrusion of the intestine. The coast in both of these conditions is sufficiently clear, so clear, indeed, that he who runs may read and accurately interpret. But it is altogether different when the abdomen has been pierced by a narrow instrument, as a knife or a dirk, or perforated by a bullet. In such an event the bowel does not protrude, and hence the true nature of the case must be solely a matter of conjecture. All that is positively certain in such event is that there is a wound in the wall of the abdomen. The surgeon, especially if called immediately or soon after the receipt of the injury, must be in doubt whether the weapon has entered the bowel or not. In reflecting upon the subject, he recalls the fact that a bullet, a rapier, a sword, or a ramrod, has occasionally passed through the abdomen, and, perhaps, even emerged at the opposite side, without, in the slightest degree, interfering with any of its contents. The records of surgery furnish many such cases.

The two principal signs which must serve to guide us in these uncertain cases are tympanites and a discharge of blood by the anus. The occurrence of tympanites is unquestionably a symptom of great value. Jobert, who was the first to notice it, regards it as the most reliable of all the phenomena when there is no escape of feces, mucus, bile or other fluid at the abdominal wound, and in this opinion the results of my personal observation fully coincide. The tympanites supervenes at various periods; sometimes almost immediately after the wound in the bowel has been received, and is then always of proportionate diagnostic value; at other times it supervenes very gradually, and in some cases, again, it does not make its appearance under twenty-four, thirty, or thirty-six hours. However this may be, it is always diffused, not circumscribed, and sometimes reaches an enormous height, the belly emitting a hollow, drum-like sound on percussion, and is then always very painful.

Although tympanites is generally present in lesions of this kind, there are cases in which it is entirely absent, as, for example, when the wound in the bowel amounts to a mere puncture, in which the opening is effectually closed by the protrusion of the mucous membrane, thereby preventing all escape of gas into the peritoneal cavity.

A discharge of blood by the anus I regard as a very valuable symptom of the existence of a wound in the bowel. It is especially valuable when it makes its appearance within a short time after the infliction of the external wound, and when it continues, more or less abundantly, for some days afterwards. As the blood is always intermixed with the contents of the bowels, it seldom comes away in a pure state, but is generally of a dark color, and of a grumous consistence.

No useful conclusions can be deduced from the shock and the pain which attend lesions of this character, since both vary greatly in different cases and in different circumstances, some persons suffering very little, while others, owing to the peculiarities of their nervous endowments, experience extreme distress.

In regard to probing wounds of this kind, the universal sentiment of the profession is opposed to it, on the ground that, while it can do no good, it would often be productive of great harm by disturbing the relation of parts, and thus endangering fecal effusion. I do not think, however, that this rule should apply to the mural wound. Here a probe, properly used, might at least afford useful information in regard to the direction and extent of the external injury.

In the treatment of wounds of the intestines two leading indications are scrupulously to be kept in view—the prevention of fecal effusion, and the occurrence of peritonitis. To secure the first, the only safeguard is efficient suturing of the wound. A case, it is true, occasionally recovers without any precaution of this kind, but this is owing to good luck rather than to good treatment. There was a time when surgeons, even of great distinction, considered the employment of sutures in wounds of the bowel utterly useless, if not absolutely detrimental. John Bell, a specious writer and an eloquent lecturer, who flourished in the early part of the century, and one of whose chief delights it was to walk roughshod over the teachings of his predecessors and contemporaries, declared that, if there be in all surgery a work of supererogation, it is the sewing up of a wounded gut. He taught that there was no peritoneal cavity, and that, consequently, there could be no fecal effusion in wounds of the bowel. The illustrious Scarpa, of Italy, for a long time the only great surgeon of that country, was equally prejudiced against the use of sutures; he considered them not only useless, but even dangerous. The practice in those days, and even at a much later period, was, in all lesions of this description, to pass a suture through the wound, and to bring out the ends at the external opening, a practice necessarily and inevitably followed by an artificial anus, and one by no means always, if indeed generally, free from the risk of fecal extravasation. The late Professor Gibson, of Philadelphia, writing in 1838, advocates a similar procedure; and Professor Syme, of Edinburgh, four years later, expressed himself to the same effect. These views may be regarded as a pretty correct expression of the teachings of surgeons in regard to this class of injuries forty-five years ago, notwithstanding the light that had been thrown upon them by Travers, Sir Astley Cooper, Lawrence, and others in England, and Jobert, Lambert, Gely, Choisy, and others in France.

The question here naturally arises, should all wounds of the bowel, however small, be sutured? Upon this subject there was certainly till recently, if indeed there is not still, some diversity of opinion. Dionis, Palfin, Heister, and Sabatier, state that enterrorhaphy is unnecessary when the wound does not exceed the diameter of a goose-quill or a penknife; and views of a similar nature are to be found in other writers, as Sharp, Richerand, Boyer, and Jobert. On the other hand, there are surgeons who are opposed to the return of the bowel into the peritoneal cavity, however small the intestinal wound, without the employment of sutures, lest fecal extravasation should ensue. The great Benjamin Bell, of Edinburgh, writing near the close of the last century, holds, in the midst of the darkness that surrounded him, the following emphatic language: "However small a wound," he says, "of the intestine may be, it ought always to be secured with a ligature, for although it is alleged by some that we should rather trust to nature for the cure of a small opening than to insert a ligature, to me it appears that the opinion is by no means well founded, insomuch that I would not leave even the smallest opening that could admit either feces or chyle to pass, without stitching it up. Much danger may ensue from omitting it, and the hazard of the patient cannot be increased by the practice being adopted."

This advice of the sagacious Scotchman, so clearly and emphatically enunciated nearly a century ago, is now the universal practice in all cases of wounds of the bowel, however diminutive, based as it is upon the well-ascertained fact that enterrorhaphy, when properly performed, is a harmless operation as compared with the risk of fecal extravasation and the consequent certainty of peritonitis.

It was with a view of testing this very matter, by determining how far even a small wound of the bowel might safely be entrusted to nature for a cure without the aid of sutures that I was induced to undertake the elaborate series of experiments alluded to in the opening part of this paper. These researches afforded abundant proof of the correctness of Mr. Bell's views; for, although I found that the wound, when it did not exceed two lines, or the sixth of an inch, was always closed by the intrusion of the mucous membrane, yet this did not always protect the parts from the effusion of fecal and other matter. Hence I have ever since laid the greatest stress, both as an author and as a public teacher, upon the importance of close suturing in all wounds of the intestines, however small. It is easy to conceive that even a very small wound, although completely occluded by the mucous coat at the moment of the restoration of the bowel, might, by the transit of stercoraceous matter, or by violent peristaltic action, become so unlocked as to admit readily of the escape of fluid or solid substance into the peritoneal cavity. Let it ever be borne in mind that the smallest possible quantity of fecal matter would be sufficient, certainly in many cases, if not in all, to light up fatal inflammation; and let this fact serve as the keynote to our practice in all cases of wounds of this description.

Judging from the results of my own observations, I have long been of the opinion that there are only two sutures that should ever be employed in sewing up a wounded bowel. These are the continued and interrupted, with the modifications of the latter by Lembert and Gely. As to Jobert's method, which consists in invaginating the ends of the bowel, when completely cut across, so as to place the two serous surfaces in immediate contact to facilitate their prompt union, the operation is not only extremely d'fficult, but very liable, even if successful, to be followed by more or less contraction of the tube at the seat of the injury, thereby interfering more or less seriously with the transmission of its contents.

The interrupted suture is, as a rule, preferable to the continued in all wounds of the bowel, whatever their extent or direction, whether they embrace the entire calibre of the tube or only a limited portion, and whether they are circular, oblique, or longitudinal. The operation executed with a long, slender sewing-needle armed with a thin but strong, well-waxed silk thread, is comparatively simple, affords ample security against fecal effusion, and is never followed by injurious contraction of the tube. The sutures should be placed not more than one line and a half, or the eighth of an inch apart, and the ends, tied in a double knot, should be cut off close, so that, in time, the sutures may find their way into the bowel and be discharged along with its contents. I deem it very important that each suture should be fully one line from the edge of the wound, and that the needle should be passed deeply through the wall of the bowel, instead of embracing its entire thickness, an arrangement which would almost inevitably be followed by more or less puckering, and by the consequent retardation of the cure. The operation of uniting the bowel where the division is complete, will be greatly facilitated if the first suture be inserted at the mesentery and the second immediately opposite.

The best, certainly the safest, ligature for suturing a wounded intestine is ordinary sewing-silk, well waxed, and inserted with a long, sharp sewing-needle. The carbolized catgut ligature is liable to give way prematurely, and should, therefore, be avoided.

In the modification of this suture by Lembert, the object is to invert the edges of the wound so as to bring the two serous surfaces in immediate and firm contact to establish, as it were, union by the first intention. Great advantage has been claimed for this form of suture, but this is not so apparent when it is remembered that, unless great care be taken in introducing it, it is liable to be followed by more or less contraction of the tube. In making this suture, the needle makes two dips on each side of the wound instead of one, as in the ordinary procedure.

"Gely's suture, which is merely a modification of that of Lembert, is made with two needles inserted near the angle of the wound, about one line from its edge; they are then carried along the interior of the bowel, parallel with the wound, for the sixth of an inch, when they are brought out precisely at the same level, so as to appear again on the peritoneal surface. The threads are then crossed, the right needle being passed through the puncture made by the left, and conversely, when the ends are firmly tied and cut off close as in the ordinary operation. The number of sutures varies, of course, according to the extent of the cut. In this way the edges of the wound are thoroughly inverted, and consequently all danger of fecal effusion is prevented; the coaptation, in fact, is so accurate as to conceal the ligatures."*

The treatment of wounds of the bowel by the continued suture has afforded good results in my experiments upon dogs. The chief objection to it is that it leaves the edges of the wound in an uneven, puckered condition, which interferes perhaps somewhat with rapid union. This, however, may be prevented in great degree, if not wholly, by the employment of a double thread after the fashion of the glover, although I do not consider this at all essential to success. Of the seventeen experiments performed with a single ligature, not one terminated fatally. The wounds in two of the cases were transverse, oblique in three, and longitudinal in twelve. The wound in one of the latter was six inches in length. The dog, a large, old one, was killed on the twentieth day, when every trace of suture had disappeared, with the full restoration of the calibre of the tube. I must not omit to state that in all these experiments the suture was passed through the fibrous tunic of the bowel, or, in other words, outside of the mucous membrane. We have here then also a very valuable suture for sewing up wounds of the intestines, especially well adapted to the treatment of longitudinal and oblique wounds; not so well, I think, to the treatment of transverse ones as the interrupted.

The suturing of the wound having been completed, and any foreign substance that may be present removed, the bowel is restored to its natural situation, followed by the omentum, in the event of its prolapse. It is hardly necessary to say that the protruded structures should be treated in the most gentle manner; any wiping that may be required should be performed with the softest cloth, and all firmly adherent matter should be picked off with the forceps. Generally speaking, the best way of cleaning the parts is to make free use of the syringe, charged with warm water. The operation may be completed with a one to one thousand solution of corrosive sublimate. The return of the bowel will be materially facilitated by the use of a little olive oil. If any serious obstacle offer, it must be surmounted with the probe-pointed bistoury, or by

*Gross's Surgery, vol. ii. p. 613, Sixth Edition.

puncture of the tube, if it depend upon the presence of gas. The wound in the wall of the abdomen should be closed in the same manner as in ovariotomy, the sutures being carried through the peritoneum so as to protect the parts effectually against hernial protrusion, a thing never to be lost sight of after such lesions.

The question arises here, what should be the conduct of the surgeon when the bowel is wounded, but not prolapsed, owing to the small size of the mural opening? I do not think that I can answer this question better to-day than I did forty years ago, when we knew comparatively little of abdominal surgery, and when the most visionary enthusiast could not have dreamed of half the triumphs that have since awaited it. The case in question is a suppositious one, and is thus stated: "A man, after having indulged in a hearty repast, receives a penetrating wound in the abdomen from the thrust of a dirk or knife; the bowel is pierced, or, it may be, nearly divided, and there is a copious discharge of fecal matter, both externally and into the peritoneal cavity, as is evinced, in the latter event, by the excruciating pain, the gastric oppression, and the collapsed condition of the sufferer. Here the most prompt and decisive measures must be resorted to, or the person will perish from peritoneal inflammation, with as much certainty as if his skull had been fractured and a portion of his brain had been let out. It will not do for the surgeon to fold his arms, and look upon the scene as an idle and disinterested spectator. Far otherwise; he has a duty to perform, and that duty consists in dilating the external wound, if it be not already sufficiently large, in hooking up the injured bowel, and in closing the solution of continuity with the requisite number of stitches, at the same time that the effused matter is carefully removed with tepid water and a soft sponge. All wiping must, of course, be carefully avoided, as this would add much to the risk of peritonitis.

"By the above procedure, which, under the circumstances pointed out, I should not hesitate to pursue, the patient is not placed in a worse condition than a female who has undergone the Cæsarean section, or a person whose abdomen has been ripped up in the first instance; recovery from both of which is not, as is well known, of unfrequent occurrence."*

It is a rule with all educated surgeons to do the work which they are called upon to perform in as complete and thorough a manner as possible, and nowhere is this precept of greater importance than

*An Experimental and Critical Inquiry into the Nature and Treatment of Wounds of the Intestines, p. 341, Louisville, 1846.

in the treatment of wounds of the intestines. A case recently reported by Professor O. K. Roberts, of Louisville. Ky., will aid me in illustrating my meaning.* A man was cut in the abdomen with a pocket-knife; the wound was three inches long; the bowel protruded, and was pierced at two points, one opening being of the size of a common lead pencil, the other of a pea. The knife in its passage had stripped off the serous membrane over a space of one inch by one-quarter. There were two slits in the mesentery, each one inch in length; and the patient had lost much blood. The mural wound was closed by sutures which embraced only the skin and superficial fascia. None of the bleeding vessels had been secured, and active bleeding was still going on from three points in one of the wounds in the mesentery, the other being occupied by a clot. It was in this condition that the man was found by Dr. Roberts, shortly after his wounds had been dressed by another surgeon. Satisfied at a glance that the case had not been properly managed, Dr. Roberts reopened the mural wound, secured the bleeding vessels with carbolized catgut ligatures, stitched the openings in the gut more thoroughly, washed out the peritoneal cavity with hot carbolized water, and closed the abdominal wound with deep sutures, completing the dressing by inserting a drainage tube in the lower angle of the wound. Under this treatment with proper subsequent care the man made a rapid recovery. Had the dressing originally applied been allowed to remain, death would have been inevitable; either from hemorrhage, peritonitis, or peritonitis and septicæmia.† The case affords a happy exemplification of hasty, careless, slovenly surgery, on the one hand, and of thoughtful, wide-awake, scientific surgery on the other.

The therapeutics after all such lesions is sufficiently simple The great point is to prevent peritonitis, or to combat it, if it takes place. The posture should be such as to relax thoroughly the abdominal muscles. The bowels should be locked up with opium to prevent peristaltic action, and nothing but iced water or pounded ice, aided, if there be much gastric distress, by a small allowance of dry champagne, should be permitted during the first three or four days. Oppression from gas should be relieved with injections of turpentine and asafœtida. Peritonitis should be met with leeching, followed by vesication with cantharidal collodion, and full doses of opium; venesection will be proper when the patient is young and robust. A laxative of castor oil, or of sulphate of magnesium, may be given at the end of five or six days, if there be

*American Practitioner, January, 1884.

marked suffering from tympanites. The urine should be drawn off during the first few days with the catheter.

I have, thus far, said nothing of gunshot wounds of the intestines. Such wounds are generally of a very serious nature, and are, therefore, liable to be followed by the worst consequences. In the first place, they are nearly always concealed wounds, from the fact that there is no prolapse of the bowel; secondly, such wounds are commonly multiple, as in one of my own cases, in which there were as many as eight perforations—two in the ileum, two in the jejunum, two in the duodenum, and two in the arch of the colon; thirdly, there is always more or less copious effusion of fecal matter; fourthly, great shock, to say nothing of hemorrhage, nearly always attends; and, lastly, most patients who survive the more immediate effects of such injuries are almost certain to succumb to peritonitis. The only rational treatment in such cases is to expose at once, or with the least possible delay, the peritoneal cavity, to stitch up, or excise, the wounded bowel, and, lastly, to clear away all extraneous matter. Excision of the tube is imperatively demanded when the wound is very large, severely contused, or very ragged. Nothing short of this would answer under such desperate circumstances; and even then no sensible surgeon would venture to pronounce a favorable prognosis.

It was my purpose, in connection with this paper, to offer some remarks on excision of the intestine in gangrene from strangulation in hernia; but it has already so far exceeded the limit originally assigned to it that I must confine myself to a few passing sentences.

The first operation that was ever performed for such an object occurred in the hands of Ramdohr, a German surgeon, who flourished in the early part of the last century. His patient was a woman, the subject of a strangulated femoral hernia, and, although fully two feet of her small intestine were removed, complete recovery ensued. This remarkable case remained for a long time a solitary illustration of the manner in which a man, inspired by genius and the gift of prevision, may project himself into the future far in advance of his plodding contemporaries. Towards the close of the last century, and the beginning of the present, operations of this kind became more frequent, chiefly through the labors and writings of Sir Benjamin Travers and Sir Astley Cooper, of London; Duverger, Boyer, and Lavielle, of France; and Schmidt and Dieffenbach, of Germany. In this country, the first case of the kind occurred in the practice of the late Dr. Charles Luzenberg, of New Orleans, in 1846. The case was one of strangulated inguinal hernia, in which that distinguished surgeon, on the basis of the recommendation contained in my monograph on wounds of the intestines, excised six inches of the ileum, and succeeded in curing his patient. Operations of this kind, dating from these humble and uncertain beginnings, now justly rank among the established resources of surgery. In an article published in *The Medical News* for March 15, 1884, Prof. S. W. Gross refers to 67 cases of this kind, in the hands of different surgeons, nearly all European, of which 21 made complete recoveries, 2 escaped death with an artificia anus, and 44, or 65 per cent., perished. This remarkable mortality seems to have been due, not, as one might, *a priori*, have supposed, to peritonitis, but to the unsound condition of the bowel beyond the seat of the gangrene, a fact of great practical value, and one that should not be lost sight of in operations undertaken for this purpose.

